# Bibliometric analysis of ovarian cancer immune evasion research from 2015 to 2024

**DOI:** 10.3389/fonc.2025.1732120

**Published:** 2026-01-21

**Authors:** Xiaodong Wang, Di Xiong, Songli Cui, Bingchen Duan, Yiping Huang, Gouping Ding, Yixuan Tang, Qianqian Wang

**Affiliations:** 1 Department of Oncology, Zhuzhou Hospital Affiliated to Xiangya School of Medicine, Central South University, Zhuzhou, China; 2 Department of General Medicine, Zhuzhou Hospital Affiliated to Xiangya School of Medicine, Central South University, Zhuzhou, China; 3 Department of Orthopaedic Surgery, Zhuzhou Hospital Affiliated to Xiangya School of Medicine, Central South University, Zhuzhou, China

**Keywords:** bibliometric analysis, immune evasion, immunotherapy, ovarian cancer, tumor microenvironment

## Abstract

**Background:**

Ovarian cancer remains lethal and shows limited response to immunotherapy partly due to immune evasion. We mapped global research trends on ovarian cancer immune evasion during 2015–2024.

**Methods:**

Web of Science Core Collection and Scopus were searched on 6 Oct 2025 for English articles and reviews published 1 Jan 2015–31 Dec 2024. Records were merged and deduplicated in R (bibliometrix). Productivity, collaboration, keywords, thematic clusters, and burst terms and citations were analyzed using bibliometrix, VOSviewer, and CiteSpace.

**Results:**

A total of 496 publications from 202 sources were included, showing rapid growth (annual growth rate ~24.6%) with a marked rise after 2020. The United States and China contributed the most output, whereas international collaboration was limited (~9.7% of authors with multiple-country affiliations). Keyword co-occurrence revealed major themes in immunotherapy, tumor microenvironment remodeling, immune checkpoint regulation, resistance mechanisms, and genetic/epigenetic modulation. Emerging hotspots highlighted tumor-associated macrophages and STAT3-centered signaling as key drivers of immune suppression and therapeutic resistance.

**Conclusion:**

Research on ovarian cancer immune evasion is expanding quickly and is shifting toward actionable targets and combination strategies. Strengthening cross-country collaboration and focusing on TME- and STAT3/TAM-directed interventions may accelerate translation and improve immunotherapy outcomes.

## 1 Introduction

Ovarian cancer (OC) is a highly lethal malignancy with a five-year survival rate below 50% for advanced-stage disease, owing to late diagnoses and frequent recurrences (1, 2). High-grade serous ovarian cancer (HGSOC), the most common subtype, is particularly aggressive and often develops resistance to standard chemotherapy (3). In recent years, advances in immuno-oncology have shifted attention toward the OC tumor microenvironment and the mechanisms of immune escape that allow tumor cells to evade destruction by the host immune system (4, 5). Immune evasion enables OC cells to avoid immune surveillance, fostering an immunosuppressive niche that promotes tumor progression and therapy resistance (5). OC tumors employ diverse strategies to blunt anti-tumor immunity – for example, recruiting immunosuppressive cells such as tumor-associated macrophages (TAMs) and regulatory T cells (Tregs), upregulating inhibitory checkpoint molecules like PD-1/PD-L1, and undergoing metabolic and epigenetic adaptations that diminish immune cell function (4, 6). Consequently, OC is often characterized as an “immune cold” tumor with low T-cell infiltration and poor responsiveness to immunotherapies (7).

**Figure 1:**
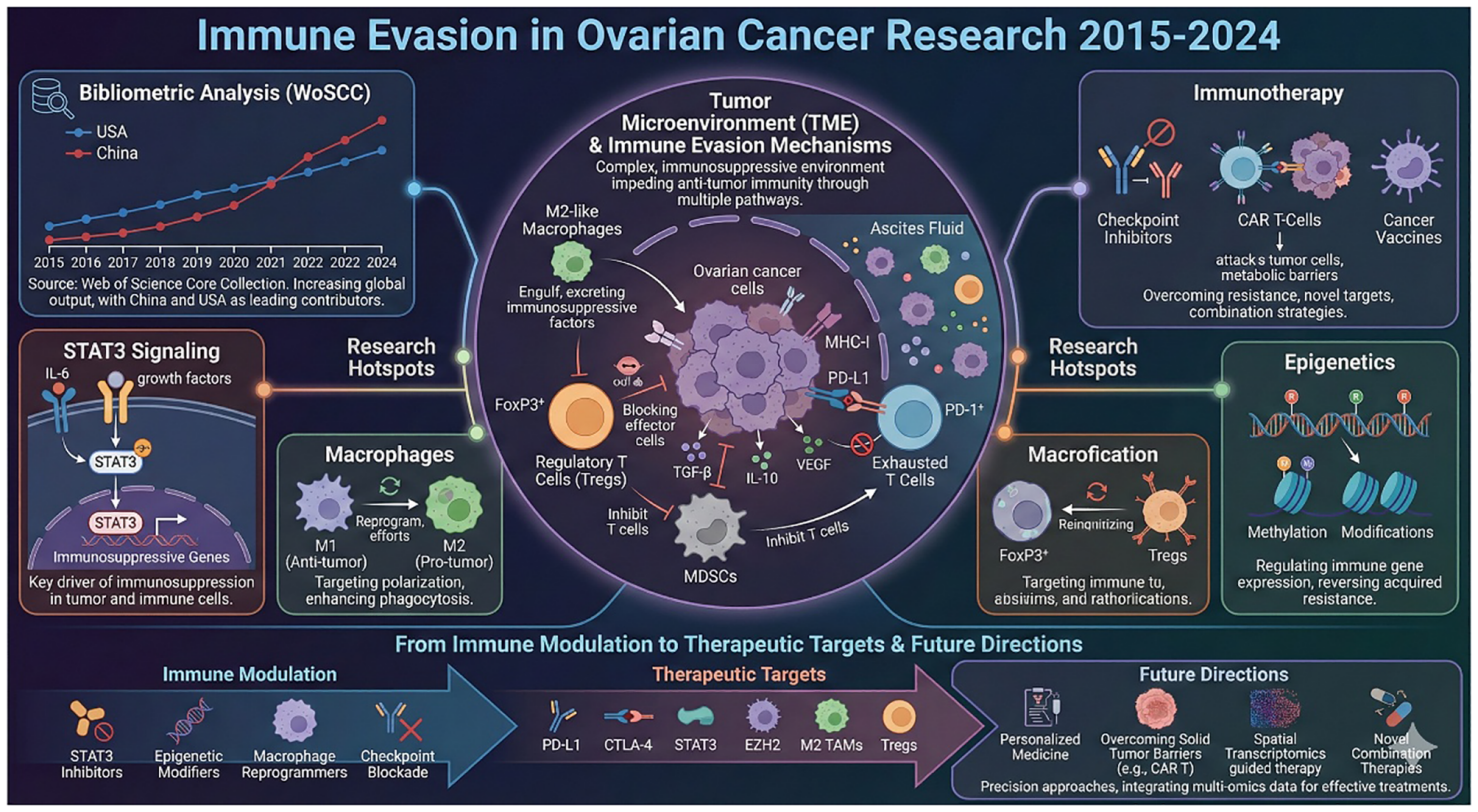
Graphic Abstract. This figure provides a visual summary of the bibliometric analysis conducted on ovarian cancer immune evasion research, covering the period from 2015 to 2024. It highlights key metrics including publication growth, emerging research themes, and the geographic distribution of research efforts.

Despite growing insights into these biological mechanisms, a comprehensive mapping of research trends and knowledge gaps in OC immune evasion has been lacking. Bibliometric analysis offers a quantitative approach to evaluate the evolution of a research field, revealing publication dynamics, thematic developments, and influential works (8, 9). Prior bibliometric studies on related topics – for instance, OC immunotherapy – have shown rapid growth in publications and hotspots such as checkpoint inhibitors and combination therapies, underscoring the value of examining the immune evasion subfield (10, 11). Here, we present a bibliometric analysis of the OC immune evasion literature from 2015 to 2024 – a period marked by breakthroughs in immunotherapies – to delineate trends in annual output, regional research contributions, collaborative networks, keyword clusters, and citation patterns. By integrating data from two major databases (Web of Science and Scopus), our study highlights emerging research foci and influential studies, providing insights into future directions for overcoming OC’s immunosuppressive barriers and improving patient outcomes (**Figure 1**).

## 2 Materials and methods

### 2.1 Data sources and retrieval strategy

**Figure 2:**
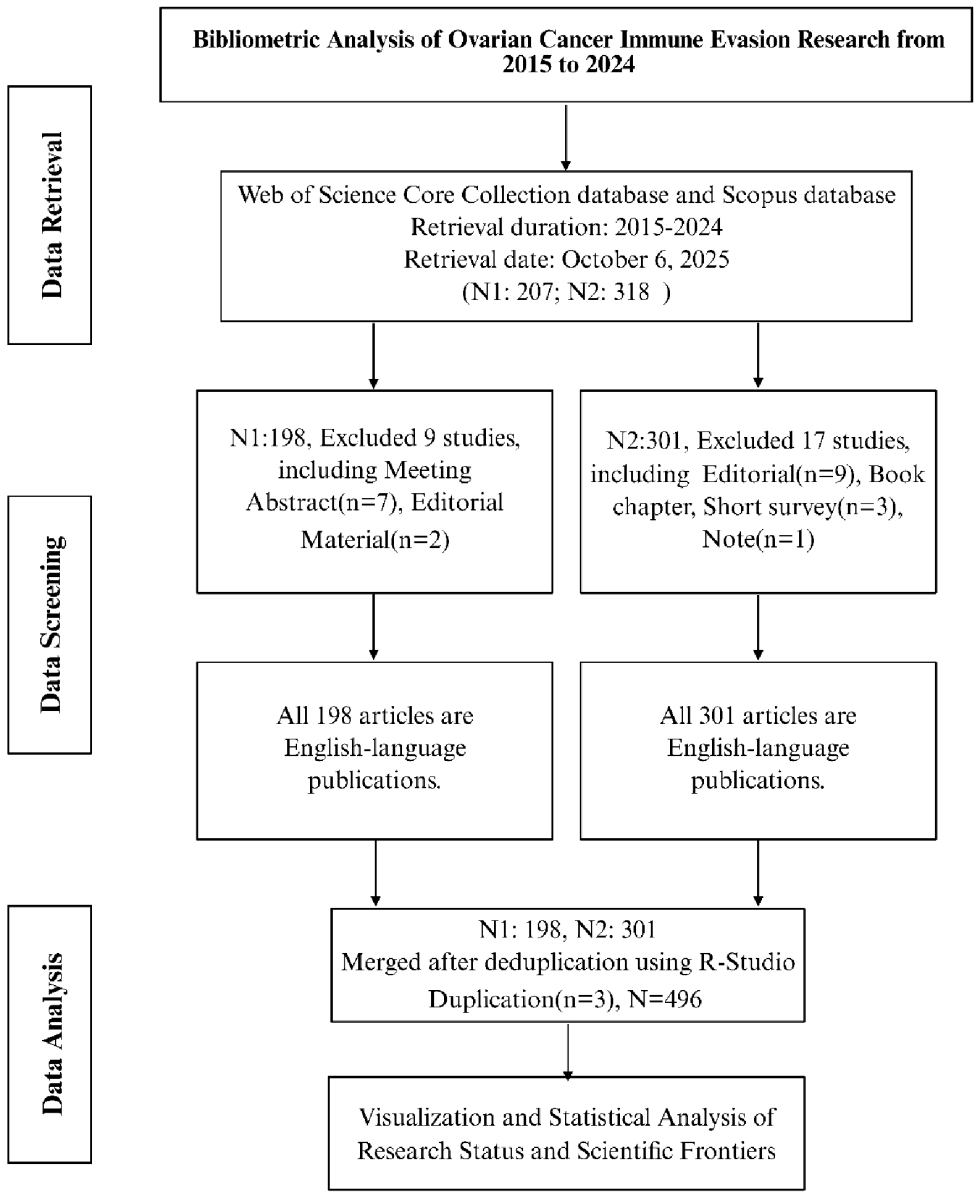
Flowchart depicting the systematic literature search and selection process. The diagram outlines the identification, screening, and inclusion of 496 deduplicated publications from Web of Science and Scopus databases, focusing on original research and review articles in English related to ovarian cancer and immune evasion, conducted on October 6, 2025. The flowchart also illustrates the methods used to ensure comprehensive inclusion and exclusion criteria, including the removal of duplicates and limitations in database selection.

We performed a comprehensive literature search on October 6, 2025, targeting publications from January 2015 through December 2024 (searching in 2025 ensured inclusion of all relevant 2024 publications). The search was conducted in the Web of Science Core Collection (WoSCC, Science Citation Index Expanded) and the Scopus database. In WoSCC, we searched the Topic, Title, and Abstract fields; in Scopus, we searched Title, Abstract, and Keywords. The search query combined terms for ovarian cancer and immune evasion, specifically: (“Ovarian Neoplasms” OR “Ovarian Neoplasm” OR “Ovary Cancer” OR “Ovarian Cancer” OR related variants) AND (“Immune Evasion” OR “Immune Escape” OR related variants). This query yielded publications discussing immune evasion in the context of ovarian cancer. Two researchers independently screened the retrieved records for relevance, and any discrepancies were resolved by discussion and consensus. We limited the selection to peer-reviewed journal articles (original research articles and reviews) published in English, excluding conference proceedings, editorials, abstracts, and other non-article documents. Records from Web of Science and Scopus were then merged, and duplicate entries (identified by matching titles, authors, and DOIs) were removed to form a unified dataset. In total, 496 unique publications meeting the criteria were included for bibliometric analysis. We focused on WoSCC and Scopus as data sources due to their broad coverage of the literature and availability of citation information, while recognizing that other databases and non-English literature were outside the scope of this study. A complete flowchart illustrating the process of identification, screening, and selection of studies is presented in **Figure 2**.

### 2.2 Data visualization and analytical techniques

We utilized several bibliometric tools to analyze and visualize the data. The bibliometrix package (v4.5.1) in R was used to merge the datasets and calculate general indicators (e.g. publication counts, citations) (12–14). VOSviewer (v1.6.20) was employed to construct and visualize keyword co-occurrence networks, revealing major research themes (15, 16). In constructing the co-occurrence network, we included keywords appearing at least 10 times to focus on significant terms. Co-occurring keywords were grouped into clusters using VOSviewer’s clustering algorithm, and a temporal overlay was applied to assess how clusters emerged over time. We used CiteSpace (v6.4.R1) to detect “burst” keywords and references that showed a sudden increase in research attention (17, 18). CiteSpace analysis was run with a one-year time slice, applying its built-in burst detection algorithm (based on Kleinberg’s algorithm) to identify terms with sharp rises in usage or citation frequency. We extracted the top 25 keywords with the strongest bursts and also identified the most prominent citation bursts among references. All analysis parameters (software versions, keyword occurrence threshold, etc.) are reported to facilitate reproducibility. Citation metrics (e.g. total citations per article) were taken from the merged database records as of the search date (October 2025) and were carefully handled to avoid double-counting for duplicated entries. All figures and network visualizations were generated with high resolution for clarity.

## 3 Results

### 3.1 Overview of publication output and collaboration

**Figure 3:**
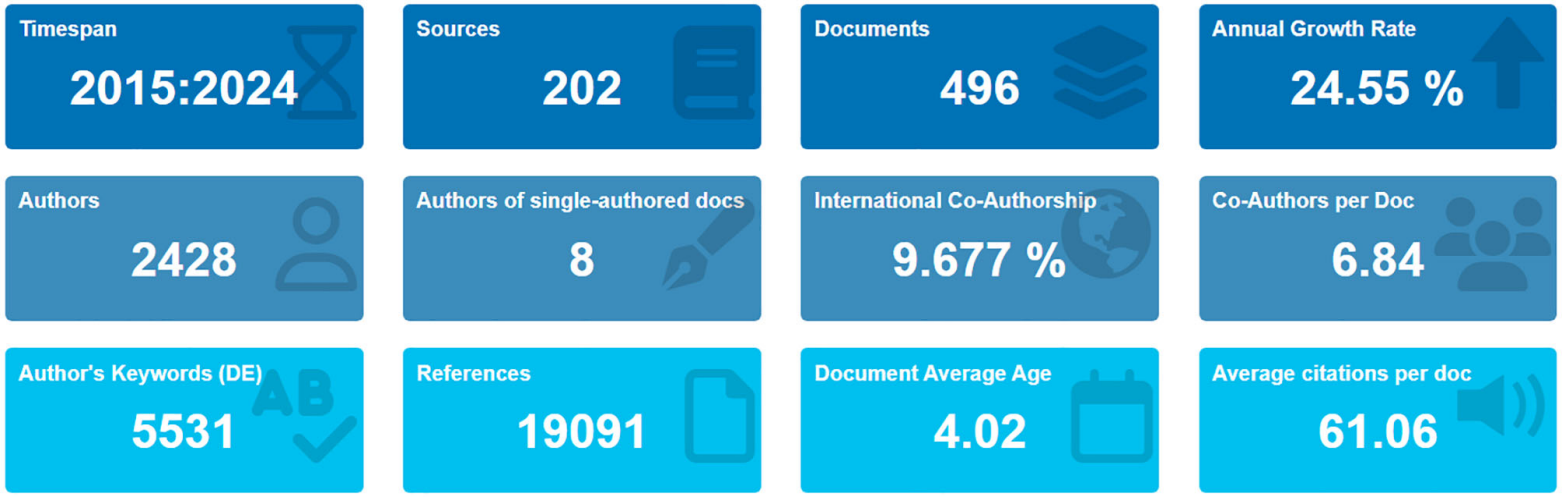
Bibliometric overview of ovarian cancer immune evasion research (2015-2024). This figure displays key bibliometric metrics such as publication output, growth rate, author collaborations, and citation patterns over the 2015–2024 period. Notably, it highlights a sharp increase in publication volume after 2020, reflecting the expanding interest in immune evasion mechanisms and the broader context of immunotherapy in ovarian cancer. The geographic distribution of research emphasizes the leading contributions from North America and Asia, particularly the U.S. and China, while also indicating regions with limited representation.

Research on OC immune evasion expanded markedly from 2015 to 2024 (**Figure 3**). Across the decade, 496 documents were published in 202 journals, yielding an annual growth rate of ~24.5%. Annual output rose from 14 papers in 2015 to 35 in 2018 and then increased sharply after 2020, reaching 101 papers in 2024. This expansion coincided with major immuno-oncology advances, including clinical adoption of checkpoint inhibitors, and with deeper insight into tumor–immune interactions that intensified focus on OC immune escape. The corpus involved 2428 authors, averaging 6.84 co-authors per paper; only 8 papers (1.6%) were single-authored, highlighting a strongly collaborative, interdisciplinary model spanning oncology, immunology, and computational expertise. International co-authorship accounted for 9.7% of documents, indicating modest cross-border collaboration and that many studies remain nationally centered, with clear scope to strengthen global partnerships.

**Figure 4:**
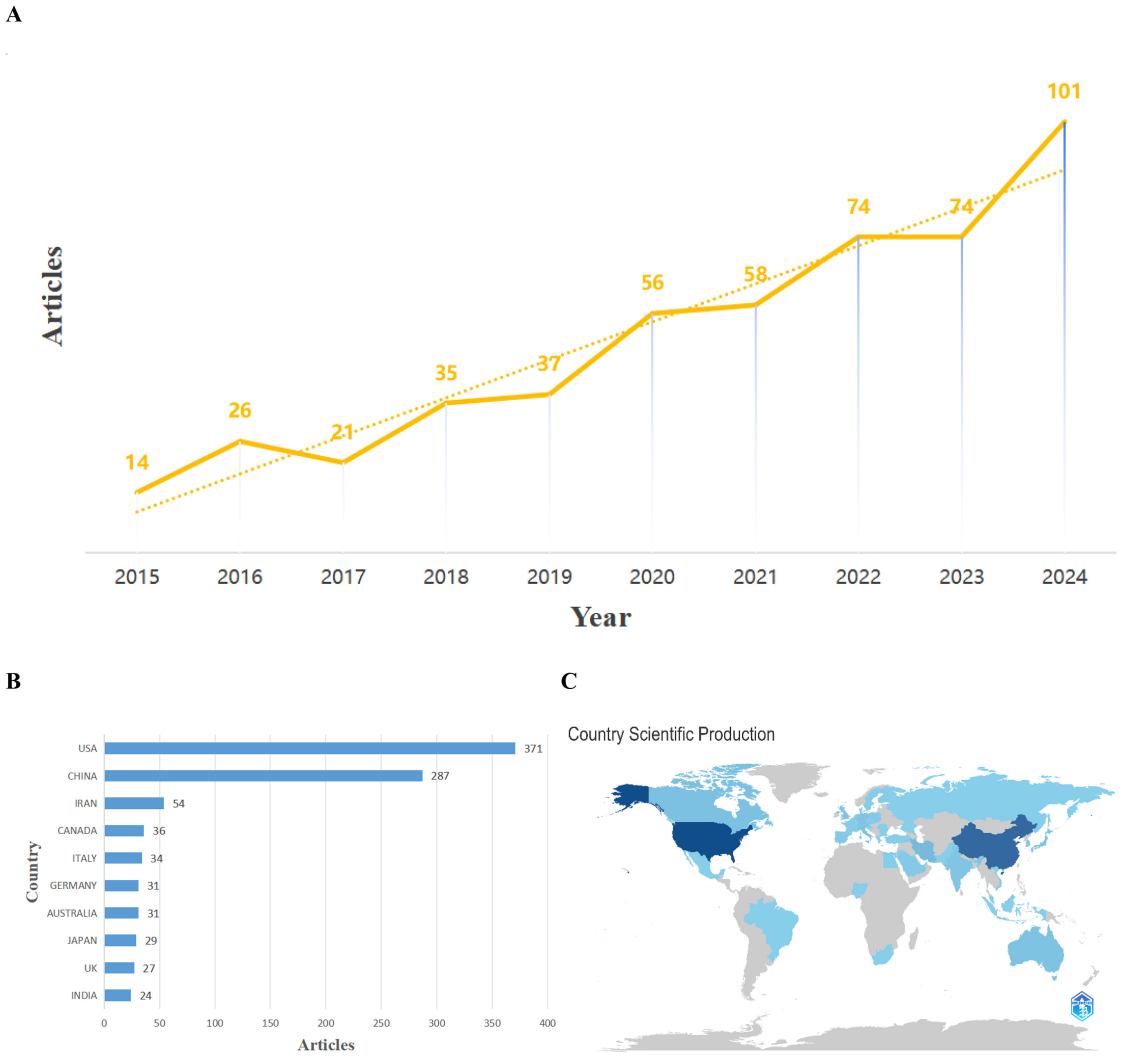
Trends in publication outputs and regional contributions in ovarian cancer immune evasion research (2015-2024). **(A)** Annual publication output overlaid with a trend line illustrating the rapid increase in research activity after 2020, coinciding with advancements in immunotherapy. **(B)** Bar chart showing the top 10 contributing countries, with the U.S. and China as dominant forces in the field. **(C)** Global map visualizing the geographic distribution of publications, with dark blue indicating high output regions and light blue signifying lower output areas. This figure underscores the need for greater international collaboration, particularly in underrepresented regions such as Africa and South America.

Geographically, output was dominated by North America and Asia. The United States contributed 371 publications (including multinational collaborations), followed by China (287). Other notable contributors were Iran (54), Canada (36), Italy (34), Germany (31), Australia (31), Japan (29), the United Kingdom (27), and India (24), with European countries contributing substantially yet trailing the United States and China. As visualized in **Figure 4**, publication density was highest in North America, East Asia, and parts of Europe, whereas most of Africa and South America showed minimal output. Few studies bridged high-output and low-output regions, underscoring underrepresentation; expanding inclusive networks and capacity-building partnerships could broaden participation and accelerate translation. This imbalance further supports coordinated funding mechanisms, shared datasets, and multicenter trials that incorporate low-output regions.

### 3.2 Temporal trends in research themes

#### 3.2.1 Publication trends

Annual output increased exponentially (**Figure 4A**). After a modest beginning in 2015–2016, publications roughly doubled by 2018 and continued to rise. A clear inflection occurred around 2020, followed by rapid acceleration: 58 papers in 2021, 74 in both 2022 and 2023, and 101 in 2024. This trajectory parallels major immunotherapy advances in oncology, including the success of immune checkpoint blockade in solid tumors and heightened attention to the tumor immune microenvironment. These developments appear to have intensified interest in OC, a malignancy prone to recurrence and therapeutic resistance. By 2024, OC immune evasion had become an established research focus, driven by efforts to define and overcome barriers that limit immunotherapy efficacy in this disease.

#### 3.2.2 Collaboration patterns

Collaboration patterns evolved modestly. International co-authorship remained low (9.7%), indicating that most groups operated within national boundaries. The United States and China, the dominant contributors, largely relied on extensive domestic networks, with some cross-national partnerships (including U.S.–China co-authored papers), but multinational studies were uncommon. **Figure 4B** summarizes leading publishing countries and **Figure 4C** illustrates geographic distribution. The prominence of the U.S. and China likely reflects substantial research investment (e.g., NIH-supported programs in the U.S.) and national cancer research initiatives in China. European contributors such as Italy, Germany, and the U.K. also published actively, often collaborating within Europe or with North American partners. In contrast, Africa and South America contributed little overall, implying constrained specialty funding and research capacity. Expanding collaborative frameworks to include underrepresented regions may reduce disparities and improve the global relevance of mechanistic insights and therapeutic strategies.

### 3.3 Keyword analysis: thematic clusters and emerging hotspots

#### 3.3.1 Keyword frequencies and co-occurrence clusters

**Figure 5:**
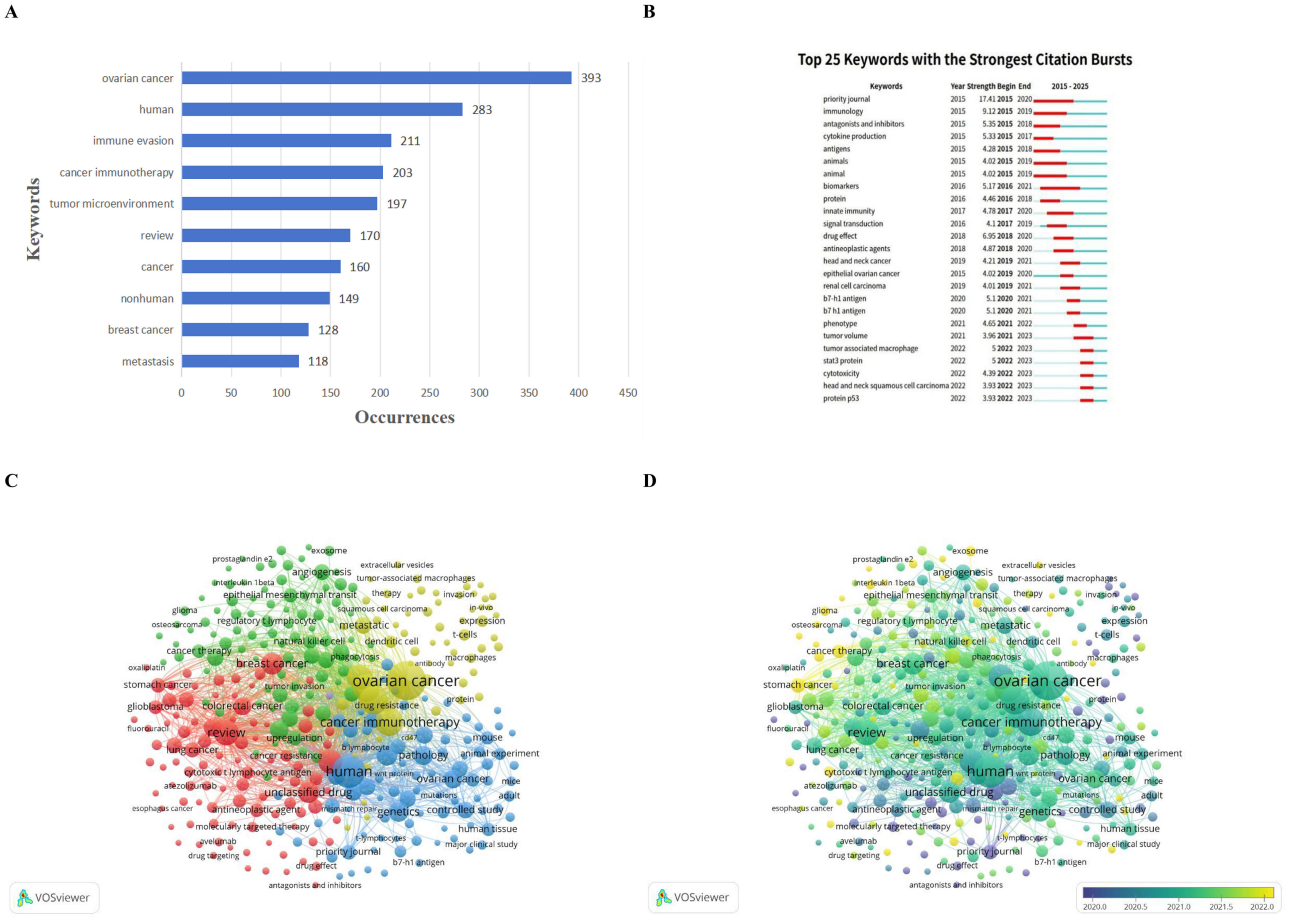
Keyword analysis in ovarian cancer immune evasion research (2015-2024). **(A)** Bar chart of the most frequent keywords by occurrence, illustrating the primary areas of focus within the field. **(B)** Timeline of the top 25 keywords with the strongest citation bursts, showing the dynamic shifts in research interest. Red bars indicate periods of heightened focus. **(C)** Keyword co-occurrence network revealing five thematic clusters of related research topics, with nodes sized by frequency and colored according to cluster membership. **(D)** Temporal overlay of the co-occurrence network, indicating how research themes have evolved over time. This analysis highlights the increasing focus on immune modulation, tumor microenvironment interactions, and resistance mechanisms as key areas of growth in ovarian cancer immunotherapy.

Analysis of author keywords (5531 in total) delineates the major themes and their interconnections in OC immune evasion research (**Figure 5**). The most frequent keyword was “ovarian cancer” (393 occurrences), confirming the disease-specific focus. Other highly frequent terms included “human” (283), indicating a strong clinical/translational orientation, and “immune evasion” (211), reflecting the field’s central construct. Immunotherapy and tumor-microenvironment concepts were also prominent: “cancer immunotherapy” (203 occurrences), “tumor microenvironment” (197), and “metastasis” (118) ranked within the top keywords, alongside broader descriptors such as “review” (170) and “cancer” (160), and comparative/preclinical tags including “nonhuman” (149) and “breast cancer” (128). This distribution suggests a literature that spans foundational immunology and model-based work to applied therapeutic research, while also leveraging shared immune-escape mechanisms described in other cancers (e.g., breast or colorectal) to inform OC-focused hypotheses and intervention strategies.

Using VOSviewer, we constructed a keyword co-occurrence network that resolved five clusters of closely related topics (**Figure 5C**). Cluster 1 (Immune Modulation and Therapy; red) grouped terms related to immunotherapy and immune-response modulation. Representative keywords included “adaptive immunity,” “antagonists and inhibitors,” and “drug targeting,” with occasional inclusion of other malignancy terms (e.g., “acute myeloid leukemia”), consistent with cross-cancer analogies in immune-therapeutic development. This cluster emphasizes pharmacologic and biologic approaches aimed at reversing immune suppression, including checkpoint blockade concepts and broader immune-modulating strategies across solid and hematologic settings.

Cluster 2 (Cellular and Molecular Mechanisms; green) centered on chemotherapy response, resistance, and delivery innovation. Terms such as “drug mechanism,” “doxorubicin,” and “nanoparticles” point to studies dissecting how drugs interact with tumor cells and the TME, how resistance emerges, and how engineered delivery platforms—particularly nanoparticle-based systems—may improve intratumoral exposure or modulate local immunity to counter immune evasion and therapy resistance. The appearance of non-OC terms (e.g., “esophagus cancer”) again suggests comparative mapping of drug-resistance or delivery principles across tumor types.

Cluster 3 (Clinical and Translational Research; blue) comprised terms linking laboratory observations to clinical application. Keywords such as “cellular immunity” and “phase 1 clinical trial” indicate that early-stage clinical evaluation of immunotherapies and combination regimens represents a meaningful component of the corpus. “Priority journal” likely reflects publication-venue tagging or meta-research emphasis that co-occurs with translational topics. Cross-cancer references (e.g., “colorectal cancer”) are consistent with shared trial designs, comparative clinical insights, and transfer of immunotherapy paradigms across solid tumors.

Cluster 4 (Genetic and Epigenetic Dynamics; yellow) highlighted foundational tumor–immune interaction biology, frequently studied in experimental systems. Key terms included “animal experiment,” “animal cell,” “gene expression,” “cytokine,” and “epigenetics,” indicating sustained reliance on *in vivo* and *in vitro* models to define how transcriptional programs and epigenetic regulation shape cytokine networks, antigen presentation, and immune recognition. This cluster captures mechanistic work in which altered expression profiles or epigenetic silencing of immune-related genes contributes to immune escape.

Cluster 5 (Tumor Microenvironment and Stroma; purple) focused on interactions between tumor cells and surrounding non-malignant components. Central terms included “immune evasion,” “immune cells,” “stroma,” “activation,” and “tumor microenvironment,” underscoring studies that interrogate how stromal composition, immune-cell recruitment, and immune activation states govern immunosuppression versus effective anti-tumor immunity. This cluster reflects the concept that immune escape in OC is shaped not only by tumor-intrinsic programs but also by the cellular and molecular ecology of the TME.

In aggregate, these clusters categorize the decade-long literature into five interlocking domains: (1) immunotherapy and immune modulation strategies, (2) cellular mechanisms of drug resistance and delivery innovation, (3) translational and clinical investigation, (4) genetic/epigenetic determinants of tumor–immune interactions, and (5) TME/stromal regulation of immune escape. Importantly, a temporal overlay of the co-occurrence network (**Figure 5D**) indicates a clear thematic shift over time. Research in 2015–2018 was weighted toward basic immunological mechanisms, including cytokine biology, immune-cell characterization, and animal-model experimentation, corresponding largely to Cluster 4 and portions of Cluster 2. By 2020–2022, emphasis shifted toward applied immunotherapy, resistance mechanisms, and microenvironmental intervention, aligning more closely with Clusters 1 and 5. This transition mirrors clinical developments: as immunotherapies entered OC trials and broader oncology practice, research focus expanded from defining immune-escape pathways to identifying actionable nodes and combination strategies that could improve therapeutic efficacy in patients.

**Table 1** provides complementary perspective by summarizing representative emerging topics within each cluster and the periods when they gained prominence. Cluster 1 showed early attention to “antagonists & inhibitors” (burst in 2015–2018), reflecting initial exploration of inhibitory pathways and immune-modulating agents; later, more advanced modalities (e.g., CAR-T-related concepts) appeared as the field diversified. Cluster 2 exhibited bursts such as “biomarkers” around 2016–2020, consistent with efforts to identify predictors of immune response or resistance, and showed increasing attention to nanoparticle drug delivery by around 2020 as nanotechnology became a widely explored approach to reshape the immune TME. Cluster 3 highlighted cross-cancer translation, exemplified by the burst of “head and neck squamous cell carcinoma” in 2022–2023, suggesting that immunotherapy insights from other tumor contexts were being imported into OC immune-evasion research. Cluster 4 included early bursts in foundational themes such as “innate immunity” (2016–2020) and sustained prominence of epigenetic concepts. Cluster 5 showed notable late bursts, including “tumor-associated macrophage” and “STAT3 protein” (both spiking in 2021–2023), indicating intensified attention to macrophage-mediated immunosuppression and STAT3 signaling within the OC microenvironment.

**Table 1:** Emerging hotspots by thematic cluster (2015–2024).

Cluster (Theme)	Representative emerging topics (Burst period or peak)
Cluster 1: Immune Modulation & Therapy	*“Antagonists & inhibitors”* (burst 2015–2018); emergence of CAR-T cell therapy and checkpoint inhibitor studies (especially post-2016).
Cluster 2: Molecular Mechanisms & Resistance	*“Biomarkers”* (burst 2016–2020); interest in *“nanoparticles”* for drug delivery (rising ~2020); mechanisms of chemoresistance (ongoing).
Cluster 3: Clinical & Translational Studies	Cross-cancer insights (e.g., *“head and neck squamous cell carcinoma”* burst 2022–2023); increasing Phase I/II immunotherapy trials in OC (notable after 2018).
Cluster 4: Genetics & Epigenetics	*“Innate immunity”* (burst 2016–2020); sustained focus on *“epigenetics”* and immune gene regulation (peaking in late 2010s).
Cluster 5: TME Interactions & Immune Escape	*“Tumor-associated macrophage”* (burst 2021–2023); *“STAT3”* signaling (burst 2021–2023); continued emphasis on TME factors (e.g., Tregs, cytokines).

This table categorizes the emerging research topics within five distinct thematic clusters based on bibliometric analysis of the ovarian cancer immune evasion literature from 2015 to 2024. The “burst periods” indicate the years in which certain keywords saw significant increases in usage, reflecting the evolving focus of the field. These clusters are crucial for understanding the shifts in research priorities, such as the growing emphasis on immune modulation therapies (Cluster 1) and the increasing attention to the tumor microenvironment and its role in immune escape (Cluster 5). The temporal trends observed in the clusters highlight the progression from basic immunology to more applied and clinical translational research, particularly after 2020.

#### 3.3.2 Burst analysis of keywords

Beyond overall co-occurrence, burst analysis (**Figure 5B**) identified keywords with sudden increases in usage, marking periods of intensified research interest and providing a timeline of trending topics. In 2015–2018, bursts were dominated by foundational or methodological themes: “priority journal” showed the strongest burst (strength 17.41; active 2015–2020), and “immunology” (9.12; 2015–2019) reflected reliance on core immunological framing. Early bursts for “antagonists & inhibitors” (5.35) and “cytokine production” (5.33), supported by “animals” (4.28), align with initial preclinical exploration of inhibitory pathways and cytokine modulation.

In 2016–2020, burst terms shifted toward clinically relevant markers and signaling biology. “Biomarkers” (5.17) and “innate immunity” (4.78) indicate intensified efforts to identify predictive markers of immune response and define innate immune roles in immune escape, while “signal transduction” (4.15) reflects increased attention to pathway-centric mechanisms (e.g., STAT3 and NF-κB signaling) that coordinate inflammation and immunosuppression.

The most recent bursts (2021–2023) illuminate current hotspots. “Tumor volume” (3.96) suggests growing interest in tumor burden as a correlate of immune contexture and treatment response. Strong bursts for “tumor-associated macrophage” (5.00) and “STAT3 protein” (5.00) confirm a surge of attention to TAM-driven immunosuppression and STAT3 signaling as tractable nodes in OC immune escape. “Cytotoxicity” (4.39; 2021–2023) highlights renewed focus on restoring effector-cell killing. Finally, bursts for “head and neck squamous cell carcinoma” (3.93) and “protein p53” (3.93) in 2022–2023 suggest increasing integration of broader oncologic concepts into the OC immune-evasion space. The persistence of multiple bursts into 2023 implies that these topics remain active and are likely to continue shaping near-term research directions.

### 3.4 Citation analysis: influential publications and reference surges

Citation-pattern analysis identified the most influential contributions to OC immune-evasion research and, through reference-burst detection, pinpointed seminal works. **Table 2** summarizes the top 10 most-cited papers, which were overwhelmingly published in high-impact venues (9/10 in Q1 journals), highlighting the clinical and conceptual relevance of this topic. The leading article was Barkal et al. (2019), “CD24 signaling through macrophage Siglec-10 is a target for cancer immunotherapy” (*Nature*), cited ~600 times (~100/year), which defined a CD24–Siglec-10 axis enabling tumor escape from macrophage-mediated clearance and proposed a therapeutically actionable target with applicability across cancers, including OC (19). The second most-cited paper, Peng et al. (2015) (*Nature*; ~1000 citations, ~100/year), showed that epigenetic silencing of TH1-type chemokines diminishes T-cell recruitment, providing a mechanistic rationale for epigenetic strategies to restore antitumor immunity in OC (20). Additional highly cited studies included Hao et al. (2019) on TGF-β–driven epithelial–mesenchymal transition and immune escape (~500 citations) and Smyth et al. (2016) in *Nature Reviews Clinical Oncology* (~800 citations) advocating TME-informed combination immunotherapy (21, 22). Collectively, the most-cited papers span checkpoint blockade concepts, immunosuppressive cytokine circuits, and metabolic/stromal constraints, underscoring the multifactorial nature of OC immune suppression. Notably, publications with lower absolute citation ranks still exhibited high annual citation velocities (e.g., Marin-Acevedo et al., 2018 on next-generation checkpoints; Anderson et al., 2017 on TME barriers to T-cell activity), indicating sustained field-wide reliance (23, 24). Across the top 10, citation rates averaged ~33–100 per year, reflecting continued visibility as the literature expands.

**Table 2:** Top 10 most highly cited publications in ovarian cancer immune evasion research (2015-2024).

Rank	Title	Publication year	Journal	JCR quartile	First author	Total citations	Citations per year
1	CD24 signalling through macrophage Siglec-10 is a target for cancer immunotherapy (19)	2019	Nature	Q1	Amira A. Barkal	600	100
2	Epigenetic silencing of TH1-type chemokines shapes tumour immunity and immunotherapy (20)	2015	Nature	Q1	Dongjun Peng	1000	100
3	TGF-β-Mediated Epithelial-Mesenchymal Transition and Cancer Metastasis (21)	2019	International Journal of Molecular Sciences	Q1	Yang Hao	500	83.3
4	Combination cancer immunotherapies tailored to the tumour microenvironment (22)	2016	Nature Reviews Clinical Oncology	Q1	Mark J. Smyth	800	88.9
5	Next generation of immune checkpoint therapy in cancer: new developments and challenges (23)	2018	Journal of Hematology & Oncology	Q1	Julian A. Marin-Acevedo	700	100
6	Obstacles posed by the tumor microenvironment to T cell activity: a case for synergistic therapies (24)	2017	Cancer Cell	Q1	Kristin G. Anderson	400	50
7	TGF-β-associated extracellular matrix genes link cancer-associated fibroblasts to immune evasion and immunotherapy failure (28)	2018	Nature Communications	Q1	Ananya Chakraborty	300	42.9
8	Host expression of PD-L1 determines efficacy of PD-L1 pathway blockade–mediated tumor regression (29)	2018	Journal of Clinical Investigation	Q1	Heng Lin	400	57.1
9	The Intersection between Tumor Angiogenesis and Immune Suppression (30)	2019	Clinical Cancer Research	Q1	Tai Hato	200	33.3
10	Canonical and non-canonical WNT signaling in cancer stem cells and their niches: Cellular heterogeneity, omics reprogramming, targeted therapy and tumor plasticity (Review) (31)	2017	International Journal of Oncology	Q2	Masaru Katoh	300	37.5

The citation counts and the citations per year data were derived from the Web of Science and Scopus databases as of October 2025. These counts may vary over time as newer publications in the field gain citations. The papers listed here represent key foundational and breakthrough studies that have shaped the field of ovarian cancer immune evasion. The distribution across top journals such as *Nature*, *Nature Reviews Clinical Oncology*, and *Cancer Cell* reflects the high-impact nature of these studies in the broader cancer immunology community. These influential papers are vital for understanding the major immune evasion mechanisms and therapeutic strategies in ovarian cancer.

**Figure 6:**
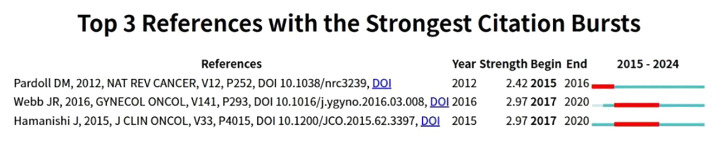
Citation burst analysis of top references in ovarian cancer immune evasion research (2015-2024). Timeline showing the three references with the strongest citation bursts, revealing key studies that have significantly influenced the field. These bursts correspond to major advancements in immune checkpoint therapies and the tumor microenvironment, marking important moments in the field’s evolution.

Reference-burst analysis (**Figure 6**) complements keyword bursts by capturing papers that exerted abrupt influence. The strongest burst was Pardoll (2012) in *Nature Reviews Cancer* (burst strength 2.42; active 2015–2016), a foundational synthesis of immune checkpoint blockade that became heavily cited as OC researchers rapidly adopted immunotherapy frameworks (25). Two additional high-burst references peaked over 2017–2020: Webb et al. (2016) in *Gynecologic Oncology* (2.97) and Hamanishi et al. (2015) in *Journal of Clinical Oncology* (2.97) (26, 27). Webb et al. linked PD-L1 expression with T-cell infiltration and favorable prognosis in HGSOC, strengthening the rationale for targeting the PD-1/PD-L1 axis (27). Hamanishi et al. reported an early PD-1 inhibitor (nivolumab) study in platinum-resistant OC, demonstrating feasibility and antitumor activity (26). The timing of these bursts aligns with the field’s transition from conceptual uptake of checkpoint immunotherapy to OC-specific biomarker studies and early clinical testing. Overall, citation dynamics indicate that the field’s intellectual core integrates high-impact conceptual advances with translational and clinical studies that catalyzed mechanistic inquiry and therapeutic innovation.

## 4 Discussion

### 4.1 Publication trends and collaborative patterns

Our bibliometric findings demonstrate vigorous expansion of ovarian cancer (OC) immune evasion research over the past decade. Publication output increased markedly after 2016 and rose most steeply during 2020–2024, coinciding with the clinical integration of immune checkpoint inhibitors and a broader recognition that overcoming immune escape is critical for improving OC outcomes. This trajectory parallels growth observed in OC immunotherapy research more generally, indicating that immune evasion has become a sustained focus area within ovarian immuno-oncology. The collaborative character of the field is evident from the high mean co-authorship per paper (approximately seven authors) and the rarity of single-author publications. Addressing immune evasion commonly requires complex and complementary methodologies, including multi-omics profiling, advanced imaging, functional immunologic assays, and increasingly sophisticated computational analyses. As a result, projects are typically executed by interdisciplinary teams spanning tumor biology, immunology, genomics, and bioinformatics, consistent with the technical and conceptual complexity of immune-escape biology.

Despite being an international research area, contributions are heavily skewed toward a few countries. The United States and China dominate publication output and together account for the majority of papers, likely reflecting substantial research funding, mature infrastructure for cancer immunology and clinical trials, and large pools of OC-focused scientific talent. European countries such as Italy, Germany, and the United Kingdom also feature among the top contributors, though at lower output levels. In contrast, Africa and South America remain minimally represented, pointing to persistent gaps in global participation. International co-authorship remains relatively uncommon (fewer than 10% of papers), and collaborations that do occur most often link established research hubs in North America, Europe, and East Asia. The limited participation of low-output regions suggests constraints in specialty funding, access to advanced platforms, and reduced integration into large consortia. Strengthening international collaboration could therefore be beneficial not only for sharing technical expertise but also for ensuring that immune-evasion insights generalize across diverse patient populations. Facilitating partnerships that include underrepresented regions—via joint conferences, consortium grants, and researcher exchange programs—could broaden perspectives, build capacity, and increase the translational relevance of discoveries. Structured consortia, shared protocols, and multicenter biobanking across regions could also reduce population bias and prospectively accelerate validation of biomarkers and combination regimens in diverse real-world settings globally.

### 4.2 Evolving research themes and emerging focus areas

Keyword analyses highlight a thematic landscape with both breadth and depth and a clear shift in emphasis over time. Early in the last decade (circa 2015), studies were largely focused on basic immunological mechanisms—characterizing how ovarian tumors suppress immune effector activity and mapping immune pathways in preclinical models. This is supported by the prominence of terms associated with foundational immunity and animal experimentation. As the years progressed, there was a clear transition toward more translational and clinical topics. By around 2020, keywords related to immunotherapeutic strategies (e.g., checkpoint inhibitors, combination therapies) and therapy resistance became more common, reflecting the practical challenges of extending immunotherapies to OC and the need to understand mechanisms limiting efficacy in patients.

The identification of five thematic clusters—immune therapy, molecular mechanisms, clinical translation, genetics/epigenetics, and tumor microenvironment (TME) interactions—underscores that immune evasion is multifaceted and interdependent rather than compartmentalized. Insights from genetics and epigenetics, including epigenetic silencing of chemokines (e.g., CXCL9/CXCL10) and downregulation of antigen presentation, have informed immunotherapy approaches that combine epigenetic priming with checkpoint blockade to enhance T-cell infiltration. Likewise, expanding knowledge about TME interactions—particularly the suppressive roles of tumor-associated macrophages (TAMs) and regulatory T cells (Tregs)—has driven exploration of agents that target these populations or their signals, often as add-ons to immunotherapy. These cross-cluster linkages illustrate how mechanistic understanding is progressively translated into therapeutic hypotheses and combinatorial strategies.

Burst keyword dynamics provide a time-resolved view of emerging hotspots. The bursts of “tumor-associated macrophage” and “STAT3” during 2021–2023 are particularly telling, indicating intensified attention to TAMs as orchestrators of an immunosuppressive microenvironment and to STAT3 as a critical signaling node in inflammation-driven immune escape. This aligns with translational efforts to reprogram or deplete TAMs (including CSF-1R–directed strategies) and to inhibit the JAK/STAT axis to reduce suppressive cytokine production, reverse T-cell exhaustion, and improve responsiveness to immunotherapy. The late-stage burst of “cytotoxicity” further suggests a shift toward restoring effector function of T cells and NK cells, consistent with the broader goal of re-enabling immune-mediated tumor killing. Together, these burst patterns imply that the field has moved from cataloguing suppressive circuits to prioritizing tractable nodes and actionable targets with potential to overcome resistance.

Another evolving focus is the metabolic–epigenetic interface of immune evasion. Beyond checkpoint pathways, OC can create a metabolically hostile environment—through elevated lactate and depletion of key nutrients such as tryptophan—that suppresses immune-cell function. Epigenetic regulation can further reduce chemokine expression and antigen-processing/presentation machinery, allowing tumors to evade immune recognition. Connecting these insights to therapeutic tactics remains challenging but increasingly central to therapeutic design and biomarker interpretation. The temporal trajectory from earlier bursts in “innate immunity” and “cytokine production” to more recent emphasis on “cytotoxicity” and pathway-specific targets suggests a maturation from descriptive immunology to intervention-oriented research aimed at restoring effective immune surveillance.

Overall, thematic evolution indicates a field becoming more mechanism-driven and clinically oriented. Early foundational work established that immune evasion is pervasive in OC and catalogued diverse escape routes. Building on that base, researchers increasingly test targeted hypotheses framed as actionable interventions—for example, whether inhibiting a defined pathway or modulating a specific suppressive cell type can restore immune control, particularly in multi-agent regimens. As single-cell sequencing, spatial proteomics, and high-plex imaging become more widely applied, the field is likely to define immune-evasion “states” and patient subgroups that can be matched to tailored interventions, thereby improving the rational design of combinations and the precision of translational strategies.

### 4.3 Influential studies and citation landmarks

Citation analyses identify influential ideas and evidence that anchor OC immune evasion research. Highly cited works include both mechanistic breakthroughs and influential reviews, indicating that progress has relied on discovery as well as conceptual synthesis. Barkal et al. (2019) had broad impact by identifying CD24–Siglec-10 signaling as a macrophage checkpoint exploited by tumors and proposing it as an immunotherapy target, stimulating interest in macrophage-directed strategies relevant to OC (19). Peng et al. (2015) provided a foundational link between epigenetic regulation and immune escape by showing that epigenetic silencing of TH1-type chemokines diminishes T-cell recruitment, thereby motivating epigenetic priming approaches to “re-awaken” antitumor immunity (20). Other frequently cited contributions span cytokine and stromal pathways, epithelial–mesenchymal transition, and rational combinations tailored to the TME, underscoring that OC immune escape arises from tumor-intrinsic, microenvironmental, and systemic influences.

Reference burst analysis complements citation counts by pinpointing publications that exerted sudden influence. The strongest burst for Pardoll (2012) during 2015–2016 reflects rapid uptake of checkpoint blockade concepts as OC investigators expanded immunotherapy trials and correlative studies and sought foundational rationale for PD-1/PD-L1 targeting (25). Bursts for Webb et al. (2016) and Hamanishi et al. (2015) during 2017–2020 highlight the importance of OC-specific clinical evidence, including links between PD-L1 expression, immune infiltration, prognosis, and early PD-1 blockade activity in platinum-resistant disease (26, 27). These bursts coincide with the field’s transition from conceptual adoption of checkpoint immunotherapy to OC-tailored clinical experimentation and biomarker-driven interrogation of why responses are limited or heterogeneous.

The citation landscape also underscores an interdisciplinary bench-to-bedside continuum. Influential papers are distributed across basic science journals, conceptual review venues, and clinical trial outlets, indicating that mechanistic results gain durable impact when linked to patient phenotypes and therapeutic responses, and that clinical signals motivate deeper mechanistic inquiry. The co-citation of sources spanning molecular signaling, immune-cell biology, and trial evidence suggests that investigators increasingly synthesize knowledge across domains to build integrative models of immune escape in OC. Across influential literature, a consistent conclusion is the need for combination therapy. Landmark studies frequently recommend or test combinations that pair checkpoint blockade with epigenetic therapy, stromal remodeling (including TGF-β pathway modulation), chemotherapy, metabolic intervention, or TME-directed approaches targeting suppressive cell populations such as TAMs and Tregs, with careful attention to identifying synergistic pairs while minimizing additive toxicity. This reflects a broad consensus that single-agent checkpoint inhibition is insufficient for most OC patients and that durable benefit will require coordinated disruption of multiple cooperating suppressive mechanisms.

In summary, citation patterns illuminate a reinforcing cycle in which conceptual breakthroughs spur trials, trial outcomes expose resistance mechanisms and heterogeneity, and mechanistic studies refine targets that inform subsequent therapeutic strategies. Landmark studies will likely remain influential, but additional breakthroughs enabled by neoantigen profiling, spatially resolved immune mapping, adoptive cell therapies, and next-generation vaccine platforms are poised to reshape the intellectual core of the literature over the coming decade.

### 4.4 Limitations and future directions

Several limitations should be acknowledged. First, our dataset was limited to Web of Science and Scopus and to English-language publications; relevant studies published in other languages or indexed in other databases may have been missed. Future analyses could broaden scope by adding databases such as Embase and regional indices and by incorporating non-English literature to provide a more global perspective. Second, terminology and indexing inconsistencies can affect retrieval and keyword-based results; authors may use different labels for similar concepts (e.g., “immune escape” versus “immune evasion”), and evolving nomenclature for pathways or therapies can shift keyword visibility. Although our search strategy attempted to capture variants, some studies may be under-retrieved. Future studies could apply text mining or natural language processing to full texts to reduce dependence on author-provided keywords.

Third, citation-based metrics have inherent temporal and database biases. Older papers have had more time to accumulate citations and therefore dominate top-cited rankings, whereas recent influential work may not yet appear prominent; citation counts are snapshots that will change over time. Database-specific differences can yield discrepant tallies for the same article, and merging records can introduce minor inconsistencies. Although consolidated citation values were used at retrieval and duplicate counting was avoided, citation values should be interpreted as a time-stamped snapshot rather than an absolute measure. Fourth, the present analysis captures quantitative patterns but does not directly assess study quality or clinical impact. Not all highly cited papers translate into practice, and not all frequently published topics lead to patient benefit; some immune-evasion mechanisms intensively studied in preclinical models may face barriers in clinical translation. Integrating qualitative assessment—such as expert review, evidence grading, or mapping bibliometric signals to trial outcomes—could help distinguish mature avenues from those with limited translational tractability.

Despite these limitations, the field map suggests actionable priorities. The thematic clusters and bursts point to multi-pronged approaches integrating immunotherapy with modulation of the TME, metabolism, and genetic/epigenetic programs. Rational combination trials that pair checkpoint blockade with TAM-directed agents or STAT3 inhibition represent a logical extension of convergent interest in these nodes, and parallel strategies incorporating epigenetic priming or stromal remodeling may further enhance antitumor immunity while addressing resistance. Our mapping also suggests underexplored opportunities, including comparatively fewer publications on neoantigens and personalized immunotherapy in OC relative to other cancers; expanding immunogenomic studies and vaccine-oriented platforms may help close this gap.

Improving global collaboration and inclusivity is another major future direction. Given the skewed geographic distribution, initiatives that support researchers in low-output regions could diversify cohorts, broaden biological inference, and enhance external validity. Concrete strategies include international funding programs that require partnerships with underrepresented countries, workshops and training fellowships to build local expertise in tumor immunology and multi-omics, and twinning models pairing established institutes with emerging centers to transfer knowledge, technology, and analytic pipelines; joint conferences and consortium grants can further accelerate such linkages. Encouraging open-access publication and data sharing can also help investigators in resource-limited settings participate and contribute context-specific insights.

Finally, bibliometric monitoring should be refreshed regularly as therapeutic modalities evolve. If the past decade emphasized checkpoint blockade and TME-targeted combinations, the next may highlight cancer vaccines, mRNA-based platforms, and adoptive or off-the-shelf cellular therapies. Continual mapping can identify emerging hotspots early, detect paradigm shifts, and signal when avenues saturate, helping the community allocate effort and resources more efficiently.

In conclusion, our analysis documents rapid growth and thematic maturation in OC immune evasion research, with increasing clinical orientation and a strong pivot toward combination strategies. By expanding data sources, harmonizing terminology, strengthening global partnerships, and integrating qualitative assessment of translational promise, future research mapping can better guide the field toward interventions that enable immune recognition and elimination of OC and ultimately improve patient outcomes.

## Data Availability

Publicly available datasets were analyzed in this study. This data can be found here: The datasets analyzed in this study were retrieved from the Web of Science Core Collection (WoSCC) and Scopus databases using the search strategy detailed in the Materials and Methods section (Section 2.1). These datasets consist of publicly available bibliographic records and can be accessed directly from the respective databases (Web of Science at https://www.webofscience.com/wos/woscc/basic-search; Scopus at https://www.scopus.com). The raw data were deduplicated and merged using the bibliometrix package in R (v4.5.1). Processed data, including keyword networks, citation bursts, and visualization files generated via VOSviewer (v1.6.20) and CiteSpace (v6.4.R1), are available from the corresponding author upon reasonable request.
